# Stage and continuum approaches in prehistoric biface production: A North American perspective

**DOI:** 10.1371/journal.pone.0170947

**Published:** 2017-03-21

**Authors:** Michael J. Shott

**Affiliations:** Department of Anthropology and Classical Studies, University of Akron, Akron, Ohio, United States of America; Universidade do Algarve, PORTUGAL

## Abstract

North American lithic analysis often assigns biface preforms to discrete, successive stages defined in Callahan’s influential study. Yet recent research questions the stage concept, emphasizing instead a continuous view of the reduction process. To compare stage and continuum approaches, their assumptions are tested in experimental replicas, including Callahan’s, and empirical Paleoindian preform assemblages. In these samples, biface reduction is a process that can be tracked and measured by continuous measures of size and reduction allometry. The process is characterized by continuous variation in the rate at which preform weight declines with preform volume. That is, weight declines at an ever-declining rate through the production process. Reduction is complex, but understood better as an allometric process than as a sequence of technological stages. “Stage” may be a useful heuristic or summary device, but preform assemblages should be analyzed in detail to reveal the continuous allometric processes that govern biface production.

## Introduction

All archaeologists know that bifaces are chipped stone tools worked on two opposing faces separated by retouched edges. The category “biface” includes finished tools—points of various defined types and/or other functional classes like knives—but also unfinished specimens that span a range of reduction from slightly modified flake blanks to nearly finished tools. This paper concerns the process of biface reduction. It evaluates two views of the process’s nature, as continuous or as segmented into real, technically determined, stages [1].

In studies of biface reduction, no consistent vocabulary of relevant terms has evolved. Here I follow usage that traces back to Muto ([2], 109), in which a “blank” is an unmodified object, a cobble or large flake, of size and form suitable for reducing to a tool. A “preform” is a blank that has undergone partial modification toward a finished form. Thus, a blank is any piece of stone that can be reduced to a tool such that “The shape or form of the final product is not disclosed in the blank” ([3], 42) except in the broadest sense. A preform “is an unfinished, unused form of the proposed artifact. It is larger than, and without the refinement of, the completed tool…thick, with deep bulbar scars, has irregular edges, and no means of hafting” ([3], 85). Finished tools are the result of the final transformation of preforms.

The study of preform assemblages can reveal the production process that begins with blanks and concludes with finished tools, thereby tracing “the evolution of the biface from a raw-material blank to a refined finished product” ([4], 180). Raw cobbles rarely were thin for their plan area, and flake blanks may only have approximated this form. Most bifaces were used to pierce or cut, so their working part usually was a tip and one or more sharp, acute-angled edges. But those working edges were only part of a larger whole that had to be grasped in the hand or secured in a haft. Generally, then, bifaces were thin for their length, width and area, which served the dual purposes of producing those tips and sharp edges and providing the heft and size for prehension to use the edges effectively. Many also were modified near the base to facilitate hafting. To reach desired sizes and forms, the biface reduction process involved a series of controlled flake removals that disproportionately decreased thickness relative to length and width, while also creating two opposing faces that converged on first crudely and then finely sinuous edges. The progress, therefore, from raw cobble or blank through preform to finished tool involved allometric reduction via thinning, edge-formation and generally finer approximation to the desired final form and size.

Reconstructing reduction sequences—the patterned ways that cores and preforms were reduced to tools—is a common goal of North American lithic analysis. Holmes [5] originated the reduction concept in the context of the Trenton Gravels debate. Following him, other archaeologists today view reduction as a sequence of essentially discrete stages [1, 6]. Ironically, though, Holmes himself thought of stages as merely heuristic devices at levels from the evolution of culture ([7], 248–249) to the production of stone tools [5], i.e. as descriptive conveniences, not fixed empirical states [8].

Whatever Holmes’s view, Callahan’s Paleoindian stage model legitimately is celebrated both for the great detail of its text and the beauty of its tool drawings. As Bennett put it, “Callahan’s interpretation of the Clovis manufacture method has been the basis of all subsequent conceptual models of tool production applied to Paleoindian groups” ([9] 29) in America North and South [10], as well as to later prehistoric industries. Callahan viewed reduction largely in sequential terms, although like Holmes he spoke also of continua ([1], 3; [11], 515 captured the ambivalent character of Callahan’s views on stages and continua). Callahan established the vocabulary that endures today in Paleoindian studies and elsewhere: North American archaeologists routinely speak of, for instance, “Stage 2” or “Stage 3” bifaces. (Callahan [1] used Roman numerals to label stages, but most archaeologists now use Arabic numerals, as Callahan later did [12]. Villa et al. ([13], 446) called their equivalent units “phases,” which seemed “less formal” to them.) Doing so, they employ Callahan’s concepts and terms and engage his assumptions about the reduction process.

The concept of stage seems intuitively reasonable, particularly from the perspective of replicators whose purposes and procedures can be organized by changes in hammer and in goals as the emerging biface approaches its desired form. For instance, Apel ([14], 129), an ardent if less nuanced advocate than Callahan, perceived stages as real entities that reflect knapper intent and sequential changes in “technique and method,” i.e., as real, well defined, and unambiguous. Bleed [15] also advocated stages, to some extent equating them with any spatial discontinuity in reduction sequences rather than with discrete technical steps or processes, all based on refit chains comprising small parts of unknown representativeness of larger flake assemblages. Yet the stage concept can embed assumptions. Bleed, for instance, assumed that continuous reduction must be unbroken in time, beginning to end occurring in one episode in one place. It also assumes the validity of stages, whose number and nature vary between replicators and analysts. One unsurprising result is considerable differences in stage assignment. Also starting from Holmes and increasing in frequency as biface-reduction modeling has expanded in recent decades a view of reduction has arisen as a continuous process better suited to analysis in quantitative terms.

To ask which of stage or continuum approaches is valid is, to some extent, a contrived question because both can be useful. Yet it is not contrived to consider which approach is best. Stages are at least analytical constructs, and may or may not be real. The stage concept possesses value as an organizing device for description and perhaps comparison between industries and reduction sequences, but in some respects is inherently ambiguous and may be needlessly restrictive. The continuum alternative also is an analytical construct, possessing its own strengths and weaknesses. Neither may be sufficient alone for comprehensive study of reduction sequences. But the stage concept is of longer standing, being firmly established in thought and practice by the 1970s to the virtual exclusion, until recently, of alternatives. Even as it increasingly is questioned, the stage view’s dominance justifies focus upon it and its assumptions [16], followed by one approach to continuous analysis of biface preform data.

### The stage approach

The stage concept assumes that stages are valid and replicable. Stages are valid if they capture or correspond to legitimate patterns of association between variables, such that all members of a stage share at least some essential qualities that separate them from other stages. The qualities may be particular technological traits, ideal values of dimensions or ratios, or at least discrete, non-overlapping ranges of metric values. If what are defined as discrete stages show weak patterns of variable association, or continuous ranges of variation and significant overlap between successive stages, then they are merely subdivisions of a complex continuum of biface reduction. In that case, the stage concept’s validity is in doubt.

Stages are replicable if all archaeologists who contemplate the same specimens would define the same number of stages that possessed the same characteristics and would assign the specimens to the same stages. If what are defined as distinct stages differ in number, nature or type between analysts, or if the same set of specimens is apportioned among defined types to significantly different degree by different analysts, then the stage concept’s replicability is in doubt.

Whatever other technological reduction sequences may reveal about the nature or reality of stages in technical or spatial terms [15], focus here is upon biface production. This is not necessarily distinct from core reduction, because cores can be progressively transformed into bifaces in the course of reduction ([17], 115). Yet the process should be similar whether cores are reduced to bifaces in the process of producing usable flakes or bifaces are the sole objective.

**Fig 1:**
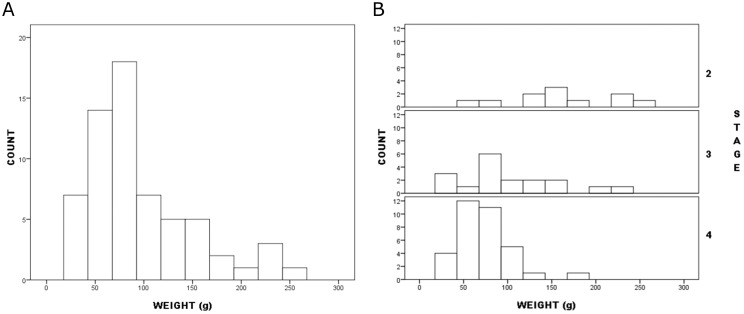
Callahan Stage 2–4 preforms frequency distribution by weight: a. all preforms; b. by stage.

**Fig 2:**
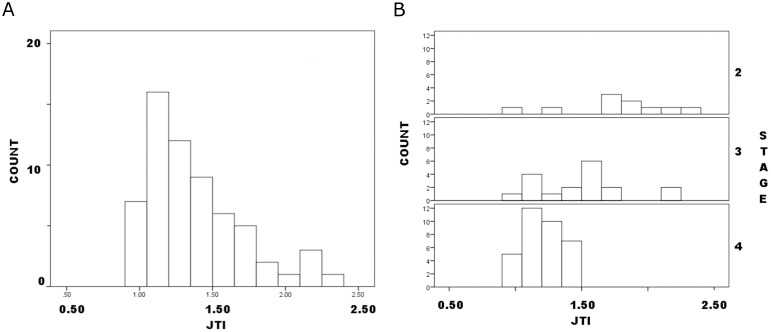
Callahan Stage 2–4 preforms frequency distribution by JTI: a. all preforms; b. by stage.

**Fig 3:**
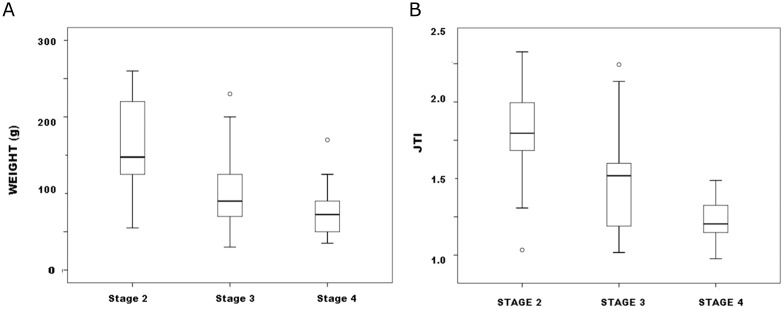
Callahan Stage 2–4 preforms boxplots by stage: a. weight; b. JTI.

**Fig 4:**
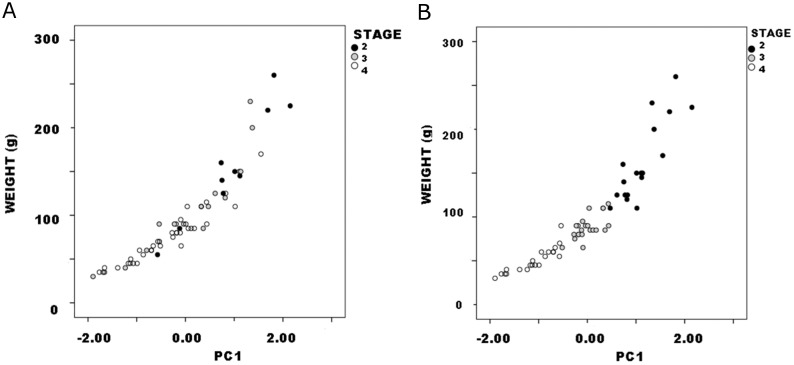
Callahan Stage 2–4 preforms, weight vs. PC1: a. actual data; b. hypothetical distribution if weight covaried with size measured by PC1.

**Fig 5:**
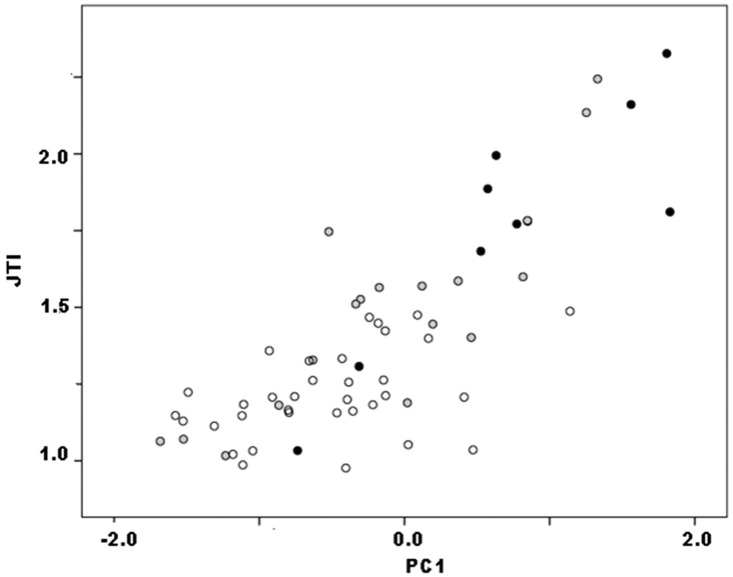
Callahan Stage 2–4 preforms, JTI vs. PC1.

**Fig 6:**
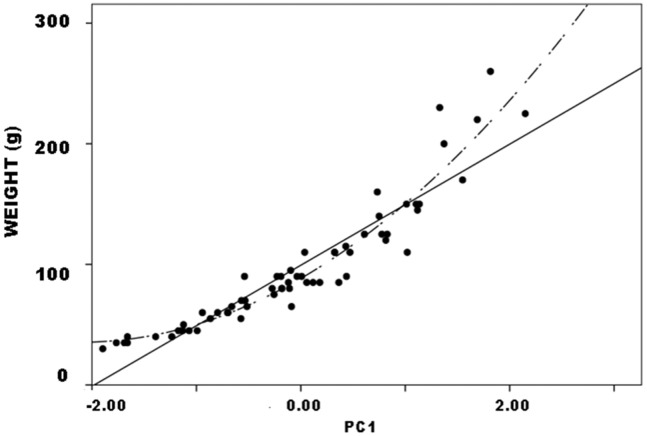
Callahan Stage 2–4 preforms, quadratic (dashed line) and Linear (solid line) Models of weight vs. PC1.

**Fig 7:**
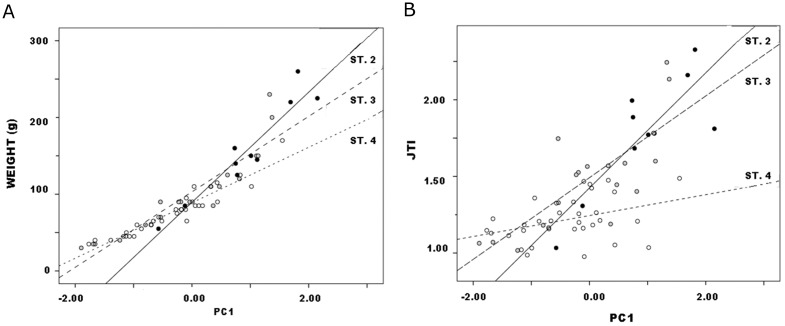
Callahan Stage 2–4 preform variables vs. PC1 by stage: a. weight; b. JTI.

**Fig 8:**
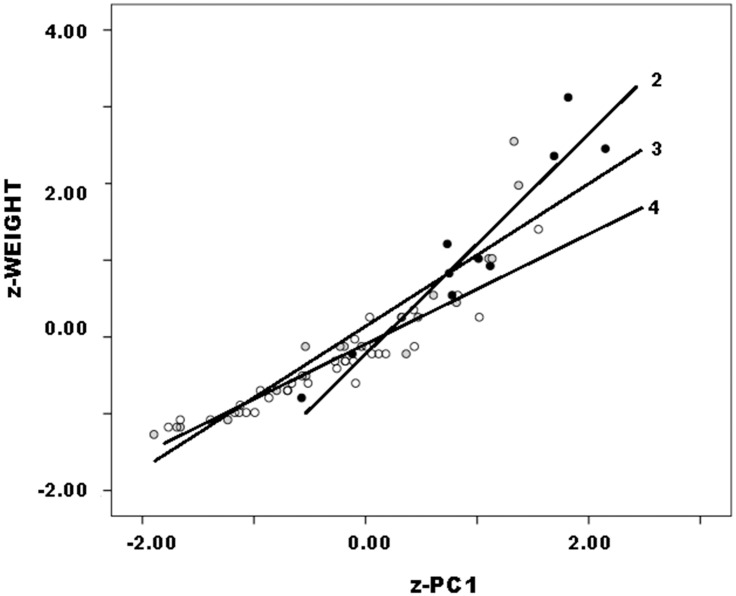
Callahan Stage 2–4 preforms, z-weight vs. z-PC1 by stage.

**Fig 9:**
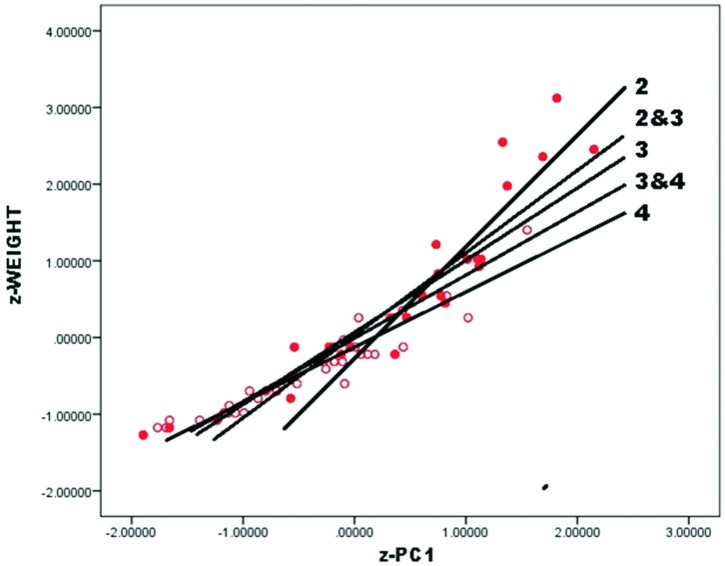
Callahan Stage 2–4 preforms, z-weight vs. z-PC1 by combined stages 2–3 and 3–4.

**Fig 10:**
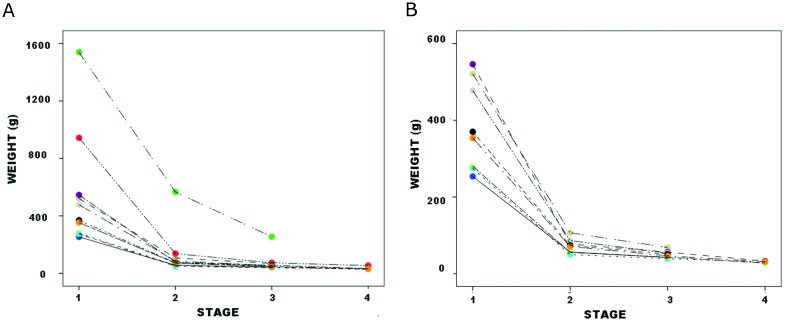
Pelegrin and Chauchat preforms, weight by stage: a. all specimens; b. two largest specimens omitted.

**Fig 11:**
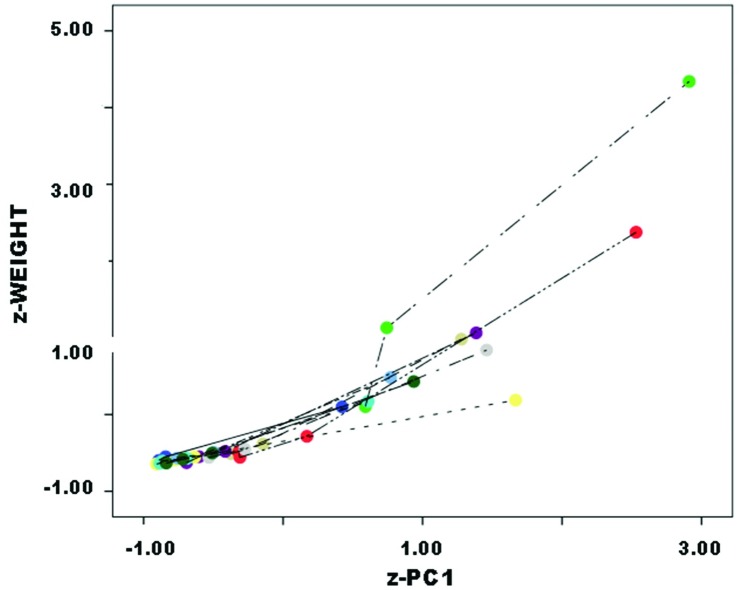
Pelegrin and Chauchat preforms, z-weight vs. z-PC1 by stage.

**Fig 12:**
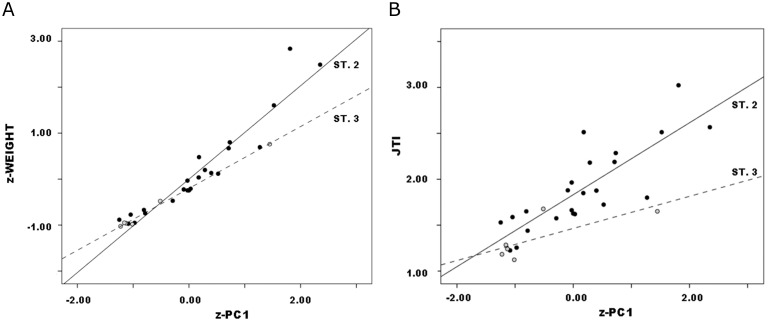
Adams Preforms, z-weight (a) and JTI (b) vs. z-PC1.

**Fig 13:**
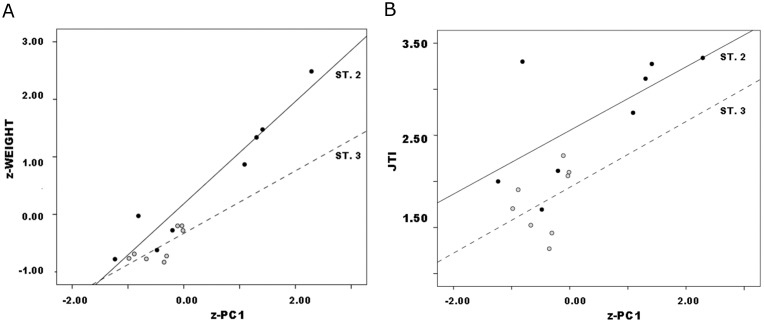
Gault Preforms, z-weight (a) and JTI (b) vs. z-PC1.

**Fig 14:**
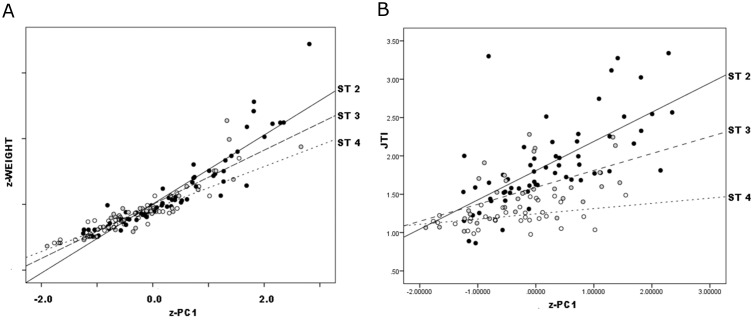
Combined dataset, z-weight (a) and JTI (b) vs. z-PC1.

Callahan ([1], Table 10) defined stages in this process by combinations of discrete and continuous variables. The former included cross-section form, flake-scar intervals, size and form of flake scars (and variation in both), extension of flakes to or across the longitudinal midline (“nature of opposing flake scar contact,” expressed as the proportion of flakes that extended to or past the midline), regularity of plan form, and platform preparation. Qualitative variables or at least criteria also included some that could only be inferred, not observed (e.g., hammer type, knapper emphasis upon edge, face, or outline, knapper concentration or attention-to-task, work pace, “degree of trim”). Interval- and continuous-scale variables included the number of flake removals, the ratio of width to thickness, edge angle, and the ratio of stage weight to final weight. In detailed description of his experimental replicas, Callahan ([1], Figs. 1–73) reported not only width-thickness ratio, average edge angle, and weight, but also length, width and thickness.

Callahan’s approach proved undeniably popular (e.g., [4], 180–181, Table 7.7; [9], 82–87; [10]; [11], 515–6; [14], Fig 2.3; [18], 26–65; [19], Table 5.23; [20], 202–214; [21], 82–110)). To Callahan Stage 1 was the blank, whether flake, core or cobble (cf. Sharrock[[22], 43] who described Stage 1 as the “flake blank” [[22], 43]] but to judge from description and illustrations [[22], Figs. 23–24] was an early-stage worked preform). Stage 2 was the interval in which initial edging of the flake blank creates the preform’s perimeter and, broadly, its plan form. In Stage 3, the preform was thinned across most or all of both faces, flakes extend across the longitudinal midline, moderately convex surfaces are formed on both faces, and edge sinuosity was reduced, all in the process of removing areas of disproportionate thickness. At this stage failure was fairly common, either by transverse fracture, plunging (*outrepassé*) terminations often called “overshots” in the Clovis literature ([[23], 68], although overshooting may be error more than design ([24], 53], and occur in later prehistoric industries as well [e.g. [17], 118]) or abandonment (e.g., remnant crowns defined by step fractures that failed to terminate normally and therefore failed to remove excess thickness). Stage 4 involved secondary thinning, yielding preforms with relatively flat faces, regular cross-sections and moderately sinuous edges. Stages 5 and higher involved largely haft (including fluted in Paleoindian industries) and perhaps tip modification.

Even Clovis-biface reduction models that differed from Callahan’s in details of fluting and finishing were descriptively similar or identical through Stage 4 ([19], Table 5.23; [20], Table 29, Fig 47). Callahan reported data only for specimens in Stages 1–4. Accordingly, handbooks of lithic analysis use Callahan’s scheme up through its Stage 4 ([4], 180; [25], 100) explicitly followed Callahan, although Andrefsky’s stage criteria did not always match Callahan’s—and the question of stages in biface reduction largely involves blanks (Stage 1) and Callahan Stages 2–4.

Table 1 summarizes Callahan’s stage-assignment scheme. Note the variability among them, and the complex nature of some of these variables (e.g., nature and size of flake scars, outline regularity) used to describe them. Perhaps as a result, in Callahan’s detailed illustrations of preforms as they progressed through his stages most discrete variables were not coded, measured, or otherwise reported. For instance, Callahan’s ([1], 68, 92, 118) Stage 2–4 exemplars were coded by degree of success, hammer type, and problems encountered—all difficult if possible at all to code in archaeological specimens—not the attributes listed in his scheme. Table 1 also summarizes biface-reduction schemes and preform stages drawn from other sources. Sharrock’s predates the Callahan model but resembles it in many salient respects; others were influenced or guided by Callahan’s approach. Muñiz’s ([17], Table 7.2) somewhat similar model emphasizes outline form, presence/absence of cortex, edging and faceting; it includes only thickness among dimensions. Huckell ([26], 191–194) also devised a similar approach that emphasized extent of flaking across the preform surface, which he considered a diagnostic Clovis characteristic (cf. [17], 122). Villa et al. ([13], 447, Table 3) defined four successive phases in production of South Africa Still Bay bifaces (excluding resharpening of finished specimens) that resembled Callahan’s stages and were defined in part by variables similar to those listed in Table 1.

**Table 1:** Callahan’s stage scheme and selected variants. Sources: [1], Fig 1–63; [4], 180–184, Table 7.7; [11], 516–518, Table 1; [18], 43–65, Appendix A; [20], 201–213, Appendices A-E; [21], 82–121, Figs 13–27, Tables 12–14; [22], 40-46b.

	Callahan	Sanders	Morrow	Sharrock	Andrefsky	Dickens	Hill
	1979	1983	1996	1966	1998	2005	2013
STAGE 1							
cortex				present			
length				50–300 mm			
w/t^1^				2			
plan form				elongate			
edge sinuosity				high			
edge angle							
cross-section							
facets				6-10/face			
STAGE 2							
cortex			present	absent		present	
length		60–112 mm				ca. 115 mm	
w/t^1^	2.0–3.0	2.0–3.0		3	2.0–4.0	2.0–3.1	2.0–3.0
plan form	irregular			elongate			irregular
edge sinuosity				lesser		high?	
edge angle	55–75°	40–110°			50–80°		55–75°
cross-section	lenticular-irreg.	lenticular-irreg.					lenticular-hexagonal-irregular
facets	12–24; variable,	none to		10-20/face			
	widely spaced,	midline	widely spaced				
	not to midline					Some across midline	not to midline
STAGE 3							
cortex				absent		rare	
length		73–137 mm		100–250 mm		ca. 104 mm	
w/t	3.0–4.0	2.6–5.5		5.0–8.0	3.0–4.0	2.1–3.5	3.0–4.0
plan form	semi-regular		ovate	elongate			semi-regular
edge sinuosity				fine			
edge angle	40–60°	40–70°			40–50°		40–60°
cross-section			regular			relatively thick	lenticular
Facets	6–12; variable,	more; more					
	closely spaced,	regular					
	across midline	pattern	across midline			across midline	across midline
STAGE 4							
Cortex				absent			
length				50–100 mm		ca. 105 mm	
w/t	4.0–5.0		4.0 ^2^	10	4.1–6.0	4.1	4.0–5.0
plan form	regular			elongate		lanceolate	regular
edge sinuosity				fine			
edge angle	25–45°	35–75°			25–45°		25–45°
cross-section	flat, lenticular						flat, lenticular
Facets	12–24; regular;						
	closely spaced,						
	across midline						
STAGE 5							
Cortex				absent			
length or size				25–50 mm			
w/t	4.0–6.0+	4			4.1–6.0	4.7	
plan form				elongate			
edge sinuosity				fine			
edge angle		40–50°			25–45°		
cross-section							
Facets							

^1^width/thickness

^2^[20], 210) reported mean length of 103 mm, width of 43 mm, thickness of 10.7 mm. 43/10.7 = 4.02.

Although there is broad agreement between schemes and fairly close agreement in some particulars, clearly these heuristic models are not entirely compatible, nor do all include the same set of measures. Some models reported dimensions by stage and employed width/thickness ratios, while others reported no size dimensions at all. Some emphasized cross-section or scar patterns; others ignored these attributes. Where it concerns flaking, some schemes referred to amount, others to pattern, still others to whether primary thinning flakes reached midlines or not. Even where specific measures like width/thickness were used, schemes differed. Andrefsky and Sharrock, for instance, used substantially different edge-angle ranges and width/thickness ratios by stage and Sanders’s Stage-2 edge-angle range was considerably wider than others’. Whatever their differences in width/thickness ratio, most sources reported progressive increase in the ratio as thickness declined disproportionately in the reduction process. Yet elsewhere Bradley ([27], Fig 17.7) reported increasing then declining ratios, such that early-stage preforms were more similar to finished points than were later-stage preforms. Archaeologists agree that there are stages to preform reduction, but apparently not always on the number or defining criteria of those stages. Clearly, there is some difference in which metric or discrete attributes are important and, in the former’s case, which values of them are important.

As above, Callahan’s stages were defined by detailed sets of discrete and continuous technological and flaking variables. Because most such variables were not reported for illustrated specimens in the original source, stage assignments using that source can produce ambiguous results. Only repeated blind tests in which several analysts independently assigned preforms to stages can determine the replicability of Callahan’s scheme. Until then, archaeologists must use their own judgment, applied to a fairly wide range of production preforms that vary not only in degree or stage of reduction but also in toolstone, flake- or core-blank size and form, and size and form of the desired end product. In the face of multiple causality Hill ([11], 515) concluded that “refined flintknapping, such as biface reduction, involved a complex decision-making process, requiring an understanding of many complex, interacting variables.” Stage models may not be the only or always the best method to determine the nature and pattern of complex variation in preform assemblages.

### Stage or continuum?

Study of any complex process, certainly of biface reduction, can engage ambiguity. To Bennett, Callahan’s approach “can be readily applied to any bifacial tool technology” ([9], 81). Yet Johnson suspected that he was “not the only one who has tried to use Callahan’s… comprehensive replicative study…only to be frustrated by the nine stage typology. It is practically like working with raw data” ([28], 159). Descriptive detail and comprehensiveness are not flaws but may act against clarity in analysis and results. Johnson legitimately questioned the workability of Callahan’s system and suggested the risk of inconsistent application of its own criteria to its subjects. Sanders found “varying degrees of Stage IV reduction” of preforms ([21], 111) at the Adams quarry/workshop, suggesting considerable variation within this “stage.” Even his analysis, which followed Callahan’s approach closely, ([21], 83,99,111) reported mean length, width and thickness values for Adams site preforms by stage that did not match Callahan’s values for the same stages. Similarly, to Amick “stage classification remains a subjective, qualitative assessment” ([29], 140).

These criticisms echo in more recent studies. Jones ([30], 23, 164) rejected stage models in part for ignoring toolstone variation that cross-cut defined stages. To Miller and Smallwood “the use of stage designations relied heavily on the lithic analyst’s subjective assessment” ([31], 31). Prasciunas found that “discrete stages in the reduction process…can be fairly arbitrary and are defined differently by different researchers” ([32], 38). The ambiguity that inheres in application of Callahan’s system is captured nicely in Dickens’s [18] comparison of the Adams assemblage [21] to his own Gault specimens. Finding significant differences in metric and other attributes in Stage 5 Adams and Gault preforms, Dickens suggested that the former “may actually be late Stage IV or very early Stage V bifaces, as opposed to a more developed and recognizable Stage V” ([18], 68).

Such views are not confined to Paleoindian studies. Despite following Callahan’s system as closely as possible and arguing for at least the partial empirical reality of stages, Beck et al. found “stage assignment…somewhat arbitrary” ([33], 494), and reported disagreement between experienced analysts in assignments. Like Beck et al., my own assignment of preforms to Callahan stages was consistent internally but not necessarily consistent with others’ ([34], 555). In Polynesian adze studies, there exists a confusing diversity in stage models resulting in a “lack of consensus in clearly defining stages along the blank-preform continuum” ([35], 362; see also [36], 10).

Accordingly, some archaeologists have questioned the validity and replicability of “stages.” As opposed to stages, even before Callahan’s influential study Muto spoke of the “‘blank-preform-product’” ([2], 109) continuum, and Collins ([6], 16–17) viewed reduction as a linear and therefore continuous process that was merely “convenient” to divide into stages. Other archaeologists demonstrated continuous variation in the reduction process, perhaps ironically by study of flake debris rather than bifaces or other reduction products themselves. Ingbar et al. ([37]; see also [38–40]), for instance, showed that reduction as reflected in the character of flake assemlages is better understood as a continuum, not a sequence of discrete stages. They accomplished this by demonstrating how flakes that were ordered by removal from cores varied not so much by stage—all in this or that stage being very similar or identical in variables that defined the stage—but by continuous degree. Even limited and incomplete refit chains from archaeological cores could be modeled in continuous terms ([16], 325–330; [41]). These flake-debris studies suggested that discrete reduction stages may exist but that they must be demonstrated, not assumed, and that the continuous nature of reduction can be modeled mathematically.

Yet continuous modeling of the reduction process came only recently to the study of production bifaces. Despite adhering to the stage view, Julig ([19], 148, Fig 5.53) plotted preform edge angle against width:thickness ratio to show a fairly strong and continuous inverse relationship that did not reveal discrete clusters corresponding to stages. To Morrow and Fiedel, “bifacially flaked artifacts represent…a continuum of all stages of fluted point production” ([42], 126), and their plot ([42], Fig 7.3) of width versus thickness of Anzick specimens showed an unbroken continuum, not clusters of values that might correspond to discrete stages. Similarly, the Murray Springs Clovis assemblage comprised a “single reduction continuum” ([26], 193). Muñiz ([17], Table 7.2) spoke of reduction in “continuum stages.” More generally in Clovis assemblages, finished bifaces “resulted from a continuous reduction process” ([23], 78). To Waters et al. at Gault “biface reduction occurs along a continuum, [so] it is difficult to assign bifaces unambiguously to specific stages” ([43], 84). Santarone recognized the need for some purposes of “imposition of categories on a reduction continuum” ([44], 21) but cautioned against concluding that imposed stages were real. Although he continued to speak of “stages” for comparison to earlier scholarship, Muñiz considered biface reduction “as occurring along a continuum” ([17], 115), the variables he coded and measured permitting him to classify bifaces and bifacial cores “based on overall morphology along a continuum of reduction” (ibid).

Consistent with these views, archaeologists have begun to develop continuous measures suitable to track variation in the reduction process. Possibly the first, Johnson’s [45] Thinning Index, is considered below. Carper ([46], 136) used a symmetry index to model continuous variation across Callahan preform stages. Wilson and Andrefsky’s ([47], 96–97) “ridge-count reduction index,” designed specifically for analysis of production bifaces rather than finished tools, and Shipton’s ([48], 151; see also ‘[49]) scar-density index expressed faceting as a function of implement size and degree of reduction. Archer and Braun’s ([50], 207) analysis of Acheulian bifaces, corroborated by experimental specimens, yielded a single dimension of continuous variation that corresponded to degree of reduction measured by number of flake removals.

One of the most significant recent studies, of the Clovis biface assemblage at Topper, questioned the validity of stage definitions and replicability of stage assignments, and argued instead that “biface production was technologically a continuous process” ([31], 29). Callahan and other stage systems yielded ambiguous results when applied to Topper bifaces. Instead, Miller and Smallwood found that continuous variables, notably a measure of average number of thinning flakes per edge that they called the Flaking Index ([31], 31)(FI henceforth), better characterized the reduction process than did assignment of bifaces to stages. Complex variation in Topper bifaces could be modeled in continuous measures; any stages defined did not pattern as discrete sets but instead conformed to continuous trends ([31], 33–34). Compared to their FI, Miller and Smallwood concluded that “traditional stage models are ill equipped for describing the variability within the biface assemblage” ([31], 36).

In recent years, then, archaeologists have expressed serious reservations about the validity of stage models. Interval- and ratio-scale measures reveal continuous dimensions inherent in the reduction process that the stage concept accommodates poorly. The diversity of views and serious doubts expressed about the stage concept, accordingly, justify its close examination.

## Materials and methods

### Testing the stage approach in continuous data

If stages exist, in Holmes’s or anyone else’s sense, analysis can reveal them. It should not, however, assume their existence. Emerging critiques of the stage approach, and the strong possibility that continuous variables can reveal nature and degree of patterning otherwise difficult to perceive, justify exploration of several related lines of continuum-based analysis. This examination is possible only because Callahan ([1], Figs. 1–63) reported weight and the basic dimensions of length, width and thickness (all in cm, converted here to mm) by reduction stages. Again, although he defined from six to nine stages, Stage 1 is the blank and later stages correspond to relatively fine details of haft-element formation, so only Stages 2–4 are considered here.

No single variable or criterion can capture nearly the full range of complex variation in biface-preforms as they progress from blank to finished production. If continuous variables reveal patterns of variation consistent with stages, to that extent a stage approach is validated. If, conversely, analysis reveals patterns of inherently continuous variation then to that extent a continuum approach is supported.

Here, Callahan’s length, width, thickness and weight serve as gross size measures and two ratios serve as measures jointly of shape and degree of reduction. One is the simple ratio of width to thickness, which figured in Callahan’s original stage approach. The other is the Johnson Thinning Index [45] devised, as in the quarry studies that partly inspired this analysis [34], to measure progressive allometric change in preforms from blank to finished biface.

Johnson ([45], 13) defined JTI as the ratio of weight to plan area:

JTI (gm/cm2) = weight / plan area

for each preform. JTI declines in value as reduction advances, because weight declines at a faster rate than plan area in the process of thinning biface preforms to completion. JTI and weight are not independent, obviously, because weight is the numerator in the ratio that yields JTI. Weight is a direct measure of size but only indirectly a measure of reduction because preforms of different weight can be at the same stage or position in reduction, and those of similar weight at different stages or positions in the reduction continuum, depending upon their starting weights and other factors. JTI is a derived reduction measure that simultaneously takes account of weight and plan area and, in the process, captures the allometry—change in shape with change in size—inherent in the reduction process.

Johnson ([45], 18) estimated plan area manually from a two-dimensional scan of each preform and polar-coordinate vectors. Amick ([29], 142) used two-dimensional scans but then a compensating polar planimeter to measure area. Like Amick, Beck et al. ([33], 495) used two-dimensional preform scans, but measured area by overlaying an orthogonal two-dimensional grid graduated in 5-mm increments on the scanned images. Thus, three different studies used different methods to calculate plan area for use in the JTI.

Here I estimate JTI (Johnson 1981:13) in a fourth way, calculated from area measured differently. The method, following Douglass et al. ([51], 518, where the expression is marred by a copy-editing error; see also [52]), models preforms as general ellipsoids whose surface areas are given from their main dimensions as:

Surface Area = [ (apbp + apcp + bpcp)/3] 1/p.

where p = ln(3)/ln(2), and a, b, and c are length, width and thickness, respectively. In [52] the expression above is multiplied by 2π to account for the three-dimensional surface area of ellipsoids. Because Johnson used two-dimensional surface area, I omitted the coefficient. In Callahan data, resulting values correlated strongly with a crude measure of plan area obtained as length multiplied by width (r = .61, p<.01), the Thomsen measure always was less than the length-width product as it must be except unless preforms were perfectly rectangular in plan form. Also, the least-squares regression intercept approached 0 and slope of the regression line of Thomsen surface area upon the product of length and width was about 0.8, i.e. <1, as it must be if the Thomsen measure varies over a narrower range than and rises with less than unit increase with the length-width product. Weight also correlated very strongly with the Thomsen measure (r = .89 p<.01). When weight was divided by the Thomsen estimate, resulting JTI values scaled similarly to values that Johnson ([45], Fig 2.6) reported.

Weight and JTI are sufficiently correlated that analysis of both may seem redundant. But all Callahan preforms analyzed were intact, whereas many archaeological preforms are broken. Weight is not a direct measure of original size in broken specimens. However, JTI is a valid measure of both intact and broken tools ([45], 18), as is FI ([31], 31). JTI scales weight to the plan area of a specimen. For instance, an intact preform that weighs, say, 10 g and measures, say, 5 cm^2^ gives a JTI of 10/5 = 2.0. If the preform were broken into two equal halves, each fragment’s JTI would be the same, 5/2.5 = 2.0.

#### Seeking stages in the distribution of continuous variables

Here I examine variation in weight and JTI by seeking gaps or modes in their continuous distributions. Then I calculate differences within and between stages in mean weight. Finally, I consider the fidelity of Callahan’s weight/thickness ratio to his stage model.

To Verrey, “Callahan’s staging hypothesis will be reinforced if there are discrete clusters of values” ([53], 4) for continuous variables like weight and reduction measures like JTI. In Callahan’s dataset, the weight distribution had a single major mode and no conspicuous gaps or secondary modes that might correspond to stages (Fig 1). The distribution was right-skewed with a very small mode at the upper extreme. But even that quasi-mode is ambiguous in distinguishing stages; of its five specimens it, three were assigned to Stage 2, two to Stage 3. Most preforms in both stages did not fall in it. Distributions by stage showed extensive overlap. Most gaps that might distinguish subsets of preforms occurred within Stage 2, not between it and other stages; such gaps may be nothing more than the product of small samples. Modes in Stages 3 and 4 were very similar in location.

The JTI distribution also was right-skewed (Fig 2); again, the highly ambiguous and very small mode at the upper end of the distribution was composed of three Stage-2 and two Stage-3 preforms, so again did not represent a discrete stage. Again, distributions by Callahan stage were similar, at least for Stages 2 and 3. Although the Stage-3 distribution included two apparent modes, one of them coincided in JTI range with the single Stage-4 mode, so preforms in the same JTI range were allocated to two different stages. On balance, no discrete modes or clusters of values were evident in the weight or JTI distributions.

When defined by combinations of categorical and continuous variables, Callahan’s stages may well be valid. But the replicability of his approach, and stages’ distinctiveness and internal integrity are undemonstrated. Mean variables by stage certainly differed (for weight, F = 13.7, p <.01; all pairwise least-significant differences [LSD] p <.03; for JTI, F = 20.7, p <.01; pairwise LSD comparisons were significant except between Stages 1 and 2)(Fig 3), no surprise since stages were defined partly by size, which these continuous variables measured. But differences in mean dimensions seem more an artifact of analysis than a property of stages themselves. Height in adult males is a continuous variable best portrayed in continuous terms. Nothing about the distribution of height in a sample of men prevents defining types or stages of height (e.g., short, medium and tall) as arbitrary intervals of the range that surely would differ in mean value as Callahan stages differ in mean size. But unless specified on valid grounds boundaries between such types are arbitrary, variation as great within as between them. There are no types of heights, but continuous variation in the variable “height.” Any types defined may impart some descriptive convenience but are not faithful to the nature of height variation.

If reduction stages are valid, then they differ among themselves as groups but specimens within a stage do not differ appreciably. This proposition can be tested in Callahan data, for instance by subdividing each stage into two equal halves (e.g. dividing Stage 2 preforms into Substage 2A, its heaviest half, and 2B, its lightest half) by weight. If stages are valid, then there should be differences between them. There are in Callahan data. But weight also differed *within* stages, i.e. between equal but arbitrary subdivisions of each stage (F = 28.1, p <.01; all pairwise LSD p <.05 except between noncontiguous Stages 2A and 4B [p = .42] and 3A and 4A [p = .26], which also demonstrates the complex, not deterministic, relationship between preform size and degree of reduction). Weight differed between successive substages as it did between stages, because both arbitrarily divide a continuum of variation. As with “types” of height, metric differences *within* Callahan’s stages were as great as those *between* them. Elsewhere, discriminant analysis using stages misclassified about 30% of Callahan Stage 2–4 specimens ([16], 321–323). Defined stages are resistant to statistical analysis.

As above, Callahan’s ([1], Table 10) stage scheme was based partly on what are reported as critical values of the width-thickness ratio. Viewing these values, arguably, as strict criteria, eight of Callahan’s 12 Stage 2 bifaces (including two broken ones not otherwise included in analysis here) fell beneath or above that stage’s range, nine of 18 Stage 3 preforms outside of that stage’s range, and that stage’s range, and 19 or 34 Stage 4 specimens outside of that stage’s range (Table 2). Thus, 38 of 64 specimens yielded width-thickness ratios that fell outside their stage’s defined range. Even if stage boundaries were broadened (for Stage 2 2.0±0.25 to 3.0±0.25, for Stage 3 3.0±0.25 to 4.0±0.25, for Stage 4 4.0±0.25 to 5.0±0.25), 28 of 64 preforms still would fall outside the specified range.

**Table 2:** Callahan preforms by stage within or beyond the stage’s width-thickness range.

	Width-thickness		n within		
STAGE	Range	n<minimum	range	n>maximum	% w/in range
2	2.0–3.0	1	4	7	33
3	3.0–4.0	3	9	6	50
4	4.0–5.0	9	13	12	38.3

### Preform analysis: Continuous variation

Distributions of individual continuous variables and ratios did not reveal empirical gaps or modes that might signify discrete stages, nor did analysis of the width/thickness ratio suggest complete validity of the stage model as conceived by Callahan. It is worth studying bivariate patterns of variation as well, either by assigned stage or across entire preform datasets. For this purpose, both the variables plotted and the form and nature of any relationship between them are continuous.

Besides ratios and weight, continuous variables that measure preform size or form include linear dimensions (minimally length, width and thickess) and weight. Here, I use a composite measure of linear dimensions produced by the ordination method principal-components analysis (PCA) of length, width and thickness. The purpose is not detailed examination of correlation among the three variables. This would discover merely the obvious, because PCA of linear dimensions typically yields a first component on which all major dimensions load positively, interpretable as a gestalt size measure (e.g., [54], 188). Instead, the purposes are first to reduce dimensions to that single size measure and second to determine if there are other significant components of variation (i.e. components whose eigenvalues>1) besides overall size.

#### PCA and regression

PCA was conducted in SPSS using the correlation matrix to minimize scale differences between linear dimensions. It produced a single significant component, PC1, that accounted for 63.3% of variance and on which all three dimensions loaded strongly and positively. As above, PC1 can be interpreted as a size measure on which all three dimensions show high positive loadings (Table 3). (See [50], 207 and [55], 142 for similar results and interpretations, using different sets of variables.) Again, it is no surprise that weight (r = .97, p<.01) and JTI (p = .83, p<.01) both correlated strongly with PC1 (Figs 4 and 5). Yet it is significant that they varied with PC1 such that lower PC1 scores and therefore smaller size indicated lower weight and JTI values. These results corroborate the interpretation of continuous PC1 as a size dimension.

**Table 3:** Summary of PCA of Callahan Stage 2–4 Data.

			cumulative	Variable		Loadings
Component	eigenvalue	%variance	%variance	length	width	thickness
1	1.900	63.3	63.3	0.797	0.826	0.764
2	0.611	20.4	83.7			
3	0.490	16.3	100.0			

Figs 4 and 5 are ordered so that weight and JTI on the one hand, and PC1 on the other both increase from the origin. Because reduction, trivially, reduces preform size, progress in the reduction continuum is read from upper right to lower left in the figures. In this context, however, the plots have three other significant aspects. First, both variables exhibited continuous distributions, not discrete clusters of observations. Continuous variables can form discrete or quasi-discrete clusters; these did not. Quadratic regression nicely described the relationship between weight and PC1 (r^2^ = .92), linear regression only slightly less so (r^2^ = .86) despite being more robust because it requires fewer parameters (Fig 6). Linear regression includes two parameters: B0, an estimate of the constant or y-intercept, and B1, an estimate of the slope of the regression line that measures the rate of change in the dependent variable with unit change in the independent. Regression is a continuous model of variation and relationship as useful in analysis of biface reduction as it was to flake debris (e.g,. [37]). Jointly in size and weight, Callahan stages comprise a continuum of variation.

Second, preforms did not sort neatly by stage in the weight:PC1 plot. Rather, there was considerable overlap among stages. Although the largest specimens were Stage-2 preforms, other Stage-2 specimens distributed across roughly three-quarters of the range in both variables and several smaller than many Stage-3 and some Stage-4 preforms. Similarly, Stage-3 preforms were almost freely interspersed on scatters. Stage-4 specimens occupied narrower ranges, but they too were distributed across roughly one-half to two-thirds of both variable’s ranges. (See [[46], Fig 8]] and [[31], Fig 3.3]] for similar plots with considerable overlap between “stages” in other preform continuous variables.) Had stages strictly segmented the continuous distributions of weight and PC1, the pattern shown in Fig 4b would be found. Jointly in size and weight, Callahan stages somewhat arbitrarily parse the continuum of variation evident in preforms.

Third, following Johnson’s (1981) logic, later stages or segments of preform finishing involve edging and thinning more than the general and size/mass reduction of earlier stages. Similarly, Smallwood’s ([56], 155–162; [57], 703) study of production-stage bifaces documented proportionally greater reduction in thickness than width in middle and late segments of the reduction continuum. No more than the progressive resharpening of finished and extensively used tools (e.g. [58]) is reduction during the production process a matter of proportional decrease such that later “stages” are scale models of earlier ones; instead it is allometric, involving change in shape and proportion with change (in stone tools, necessarily reduction) in size. Particularly significant is that regression’s B1 coefficient measures the slope, and therefore rate, of covariation between weight or JTI and PC1 (Fig 7). Following this logic, closer examination of these relationships is warranted.

#### Covariation of weight and JTI with PC1

Table 4 reports specimens by assigned stage in Callahan’s replications. Least-squares regression of weight and JTI upon PC1 was conducted separately by defined stage. Unless otherwise indicated, in this and all subsequent analysis regression results were significant (i.e. p≤.05) and slope coefficients declined by stage. Differences in regression coefficients were evaluated by Student’s t using the method of Statistical Consulting Group [59](Table 5). Slope differences therefore are relevant for their magnitude and, being consistent in direction, t values are reported without sign.

**Table 4:** Preforms by Callahan Stages 2–4 in study datasets.

	Count	by	Stage	
Dataset	2	3	4	Sources
Callahan replications	10	18	34	[1]
Pelegrin & Chauchat	10	10	8	[62]
Adams	23	6	0	[21]
Thunderbird	20	0	0	[53]
Gault	8	8	0	[18, 43]

**Table 5:** Summary of test of similarity in B1 coefficients between Callahan stages.

	WEIGHT		WEIGHT		WEIGHT	
			Callahan	Stage 2	Callahan	Stage 3
Dataset	stage	B1	t	p	t	p
Callahan	2	73.6	---	---		
Callahan	3	57.9	*2*.*08*	*0*.*05*	---	---
Callahan	4	46.8	*5*.*92*	*<*.*01*	*2*.*55*	*0*.*01*
	JTI		JTI		JTI	
			Callahan	Stage 2	Callahan	Stage 3
Dataset	stage	B1	t	p	t	p
Callahan	2	0.39	---	---		
Callahan	3	0.32	0.74	0.46	---	---
Callahan	4	0.1	*4*.*13*	*<*.*01*	*3*.*72*	*0*.*01*

Regression results for Callahan’s Stages 2–4 data, with standard errors of estimates in parentheses, are (Fig 7b):

Callahan Stage 2 *weight* = 101.3 + 73.6 * *PC*1

            (6.3) (5.8)

Callahan Stage 3 *weight* = 110.1 + 57.9 * *PC*1

            (3.8) (4.4)

Callahan Stage 4 *weight* = 99.8 + 46.8 * *PC*1

            (1.7) (2.1)

Weight patterns clearly, and slope coefficients differ significantly (Table 5), with PC1 by stage. This finding implicates reduction allometry, “stages” patterning differently such that weight decreases with size (or decreases during reduction, to read Fig 7b from right to left) at lower rates in successive “stages”. That is, weight declines with size at ever-declining rate by “stage,” i.e., arbitrary subdivision of the reduction continuum. This is no trivial statistical observation. Rather, the slope of the regression of weight upon PC1 becomes a ratio-scale estimate of degree of reduction in ranges or segments of the reduction continuum, an estimate that complements and expands ordinal-scale stage classification. Jointly in size and weight, continuous analysis of Callahan stages captures the allometry that governs biface reduction, and B1 serves as the allometry coefficient. Useful concepts like “tempo” of reduction ([57], 695, 703–705; [60], [61], 653;) measure the same process although as reported they reduce continuous variation to ranges of values that correspond to assigned preform stages.

Results for JTI are somewhat ambiguous. As above, JTI is not independent of weight (r_s_ = .90 p<.01). Nor, obviously, is it independent of PC1 which summarizes the scores of the same major dimensions that form JTI’s denominator. Regression of JTI upon PC1 by stage patterned consistently (Fig 7b):

Callahan Stage 2 *JTI* = 1.43 + 0.38 * *PC*1

          (.11) (.09)

Callahan Stage 3 *JTI* = 1.49 + 0.27 * *PC*1

          (.05) (.05)

Callahan Stage 4 *JTI* = 1.24 + 0.07 * *PC*1.

          (.03) (.03)

However, slope coefficients did not differ significantly between Stages 2 and 3 (Table 5). Moreover, for Stage 4 the scatter was diffuse, the resulting slope extremely low; although results are significant, they do not account for most variation (r = .44, r^2^ = .19, p = .01). JTI imperfectly models the reduction process in these data.

#### z-score transformations

Callahan and other datasets to which it is compared below scale somewhat differently in the range of original variables, which complicates direct comparison of their regression results. One reason is that Callahan data span Stages 2–4, other datasets only two stages, usually 2 and 3. The range of variation therefore is somewhat greater in Callahan’s than other datasets. To minimize scale differences that can affect PCA, data were transformed to standard scores (“z-scores”) for each dataset separately from others. z-scores rescale raw data to units of each variable’s standard deviation, their sign indicating that they are above (positive) or below (negative) the mean. Standardized weight is denoted z-weight. Because PCA proceeds by standardizing variables, component eigenvalues and variable loadings are identical to PCA of original variables, and the single resulting significant component is denoted z-PC1. JTI is a ratio between two original variables, thus a derived variable. JTI may be standardized as was weight, but interpretation of a standardized ratio is unclear. Therefore, JTI was not standardized, so patterned and scaled with z-PC1 exactly as it did with PC1.

As in original data, PCA of Callahan data yielded a single significant component on which z-length, z-width and z-thickness all loaded strongly and positively, so again is a general size component. Callahan’s Stages 2–4 data patterned consistently as follows (Fig 8):

Callahan Stage 2 *z* − *weight* = −0.14 + 1.38 * *z* − *PC*1

             (.20) (.16)

Callahan Stage 3 *z* − *weight* = 0.13 + 0.94 * *z* − *PC*1

             (.10) (.10)

Callahan Stage 4 *z* − *weight* = −0.15 + 0.69 * *z* − *PC*1

             (.10) (.04)

Such that regression intercepts approached or fell within one standard error of 0 and slope declined with stage as reduction progressed. All B1 coefficients differed significantly from one another (for Stages 2 and 3, t = 2.20 p = .04; for Stages 3 and 4, t = 2.61, p = .01). Trivially, Callahan z-weight against z-PC1 scaled differently from original variables; more significantly, transformed variables patterned as did original ones and yielded equally significant regression results. As above, because JTI was not standardized and PC1 and z-PC1 are identical, there is no need to recalculate the regression of JTI upon z-PC1. Also because JTI was not standardized, here and in subsequent analyses its regression intercepts considerably exceed 0.

At first glance, results might be thought to support the existence of stages because slope coefficients differed significantly between stages in regression of z-weight upon z-PC1. But preceding analysis of univariate distributions and ratios and of bivariate plots suggested instead that statistical difference may owe to stages’ arbitrary parsing of a reduction continuum. As in earlier analysis, therefore, the validity of stages must be tested in multivariate data.

Subdividing each Callahan stage as in preceding analysis is questionable, as it would involve multivariate analysis of very small data subsets. Instead, if reduction is continuous then overlapping subsets of stages should pattern in regression of z-weight upon z-PC1 score such that B1 should differ between the combinations and in value fall between the B1 values of individual stages. To test these expectations, Callahan preforms were combined into two overlapping subsets, Stages 2 and 3, and Stages 3 and 4. z-scores were calculated separately for each subset and then ordinated by PCA. As expected, B1 coefficients of the combined Callahan subsets patterned consistently, a higher value and slope characterizing combined Stages 2 and 3 (Fig 9). Slope coefficients differed significantly from one another (for combined Stages 2–3, B1 = 1.06; for combined Stages 3–4, B = 10.83; t = 2.57, p <.01), despite the two subsets sharing 18 Stage-3 specimens, and coefficients fell between the B1 values of the respective individual stages. Significant difference between stages or stage groups can occur even when such stage groups share many specimens. This conclusion implicates meaningful variation within and between “stages” that only a continuous approach to biface production can reveal.

### Other datasets

Callahan’s [1] data by stage were not linked to specific bifaces such that each specimen can be traced through the reduction continuum. Lacking this degree of experimental control, it is unclear if pattern in continuous data reflects each preform’s reduction trajectory or is the result of data aggregation. Datasets in which the same specimens can be followed through successive reduction stages should be examined for possibly similar patterning.

For instance, Pelegrin and Chauchat ([62], Tables 1–2) reported all relevant variables for experimental replicas of Paiján points, a late Pleistocene type found on the central Andean coast. Although the finished point is stemmed, their replicated specimens remain unstemmed preforms through the stages analyzed here. Pelegrin and Chauchat ([62], 370) defined sequential Stages 1–4 that followed the flake blank (*soporte inicial*). Thus, their flake blank is Stage 0 whereas Callahan’s blank is Stage 1. More importantly, individual specimens can be followed across all stages. If analysis of Pelegrin and Chauchat data resembles Callahan’s then patterning in the latter is corroborated and the approach is worth applying to archaeological data.

Pelegrin and Chauchat made 13 preforms, 10 of whose dimensions they reported across three stages, only six of them to the fourth stage ([62], Table 2, where data from subdivided Stage 2 are from Stage 2b); they reported dimensions for two additional specimens at their fourth stage, for stage totals of 10, 10 and eight preforms. Fig 10 plots weight against stage for Pelegrin and Chauchat data; because the two largest specimens are disproportionately large at Stage 1, Fig 10b omits them to more clearly show patterning in other specimens (although they were retained for all analysis reported below). All preforms followed the same pattern. As in Callahan data, PCA of Pelegrin and Chauchat’s ([62], Tables 1–2) Stages 2–4 data yielded one significant component on which z-length, z-width and z-thickness all loaded strongly. z-weight plotted against z-PC1 by specimen (Fig 11) yielded slope coefficients that ranged from 0.32 to 1.68. (Each regression involved only three data points, which for most analytical purposes are too few to lend much confidence to results. Instead, regression serves merely to corroborate patterning in Callahan data using a dataset where each preform’s dimensions and weight can be traced across “stages.”) Callahan Stage 2–4 values fall comfortably within this range, which suggests robust patterning between datasets. JTI was not calculated for Pelegrin and Chauchat specimens.

For further comparison to Callahan’s data, therefore, analysis can include Chauchat and Pelegrin’s as well as relevant North American Paleoindian datasets (Table 4). The latter include:

1. Adams. Sanders [21] analyzed the Adams Paleoindian assemblage from western Kentucky. Adams was a Clovis workshop adjacent to outcrops of Ste. Genevieve chert. Sanders closely followed Callahan’s stage scheme, at least through the Stage-4 data analyzed here ([21], 82–121), and reported length, width, thickness and weight of Stage 2–4 preforms ([21], Tables 10–12). Unfortunately, no Stage 4 preforms and only six of more than 20 Stage 3 preforms were intact, so Sanders’s data are mostly from Stage 2.
2. Thunderbird. Verrey [53] described a late Paleoindian cache, apparently of local jasper, from Feature 17 at Thunderbird. He considered “all but 3” ([53], 4) to be Callahan Stage-2 bifaces and reported 23 specimens including two biface/scrapers and one large lateral fragment, as well as metric data on cache specimens (R. Verrey, personal communication 18 November 1985). I assume that the latter three items were not Callahan Stage 2 preforms, so confine analysis to the other 20 preforms as Callahan Stage 2 specimens.
3. Gault. As above, Dickens [18] assigned Gault preforms to Callahan stages. For 16 specimens that fall in Callahan Stages 2–4, those assignments can be cross-referenced to Waters et al.’s ([43], Table 20) data.

All datasets appear in S1 Table. Other sources consulted could not be included. Despite its extensive documentation [23, 30, 63, 64], Anzick’s involved collections history prevented its inclusion. Several cataloguing systems complicated the process of reconciling specimens and their metric data between sources. Although the Anzick preform assemblage is large, relatively few retained all dimensions necessary for analysis here. Kilby could not weigh specimens, so estimated weight as a function of major dimensions, reasonable but not strictly equivalent to the direct measurement of weight in other assemblages. Jones ([30], 164) considered some Anzick specimens to be finished and used tools, not preforms. Morrow’s ([20], Appendices A-E) highly detailed dataset did not include weight. Bamforth ([24], Table 4.2) reported length, width, thickness and several ratios but not weight or stage assignment for Mahaffy preforms. Bement ([60],Table 5.1) reported all relevant dimensions and weight for JS cache preforms, but not stage assignments in Callahan’s or other schemes, nor did Jennings ([61]) report specimen dimensions or Callahan stage assignments. Muñiz ([17], Table 7.1) also reported dimensions but not weight for CW cache preforms. Although advocating a continuum-based view, he also devised a classification system different from Callahan’s, based on the treatment of preforms as cores. Muñiz assigned all 11 CW preforms to a single stage, his “late-stage bifacial core” ([17], 116). Huckell [65] did not report either dimensions or weight for the Beach cache. As useful as these sources are, none provides data that are directly comparable to Callahan’s or others’ used here.

### Analyzing combined data

#### z-weight

As in Callahan data, PCA analysis of each separate source yielded a single significant component on which all dimensions loaded positively so is interpretable as gestalt size, all regressions by stage were significant and, where relevant, successive stages had lower slope coefficients. Adams and Gault Stage 2–3 preforms showed similar patterns to Callahan data (Figs 12 and 13; Table 6). Overall, therefore, patterning was robust and clear: as reduction progressed, z-weight declined with z-PC1 at ever-declining rate. Again, this pattern was revealed only in continuous data, and itself is a continuous relationship that exclusive focus on qualitative variation would not reveal.

**Table 6:** Summary of test of similarity in B1 coefficients between Callahan stages and other data sources. Italics and underscores indicate t and p values significant @ .05, also shaded for ease of reference.

		z-weight		z-weight		z-weight		
			Callahan	Stage 2	Callahan	Stage 3	Callahan	Stage 4
Dataset	stage	B1	t	p	t	p	t	p
Callahan	2	1.37	---	---				
Callahan	3	0.94	$\underline{2.20}$	$\underline{0.04}$	---	---		
Callahan	4	0.69	$\underline{4.33}$	$\underline{0.00}$	$\underline{2.61}$	$\underline{0.01}$	---	---
Adams	2	1.02	$\underline{2.23}$	$\underline{0.03}$	0.63	0.54	$\underline{4.13}$	$\underline{0.00}$
Adams	3	0.69	$\underline{3.57}$	$\underline{0.04}$	1.39	0.17	0.20	0.84
Thunderbird	2	0.97	$\underline{2.77}$	$\underline{0.01}$	1.04	0.30	1.49	0.11
Gault	2	0.89	$\underline{2.58}$	$\underline{0.02}$	0.32	0.75	$\underline{2.49}$	$\underline{0.02}$
Gault	3	0.54	$\underline{2.33}$	$\underline{0.04}$	1.03	0.32	0.78	0.44
P&C^1^	2	1.30	0.21	0.84	1.47	0.15	$\underline{3.72}$	$\underline{0.00}$
P&C	3	0.62	$\underline{4.86}$	$\underline{0.01}$	$\underline{2.22}$	$\underline{0.04}$	1.06	0.30
P&C	4	0.18	$\underline{4.35}$	$\underline{0.01}$	$\underline{2.49}$	$\underline{0.02}$	$\underline{3.62}$	$\underline{0.00}$
		JTI		JTI		JTI		
			Callahan	Stage 2	Callahan	Stage 3	Callahan	Stage 4
Dataset	stage	B1	t	p	t	p	t	p
Callahan	2	0.38	---	---				
Callahan	3	0.27	0.23	0.82	---	---		
Callahan	4	0.07	$\underline{4.33}$	$\underline{0.00}$	0.43	0.67	---	---
Adams	2	0.39	1.46	0.16	1.62	0.11	1.93	0.10
Adams	3	0.18	$\underline{5.73}$	$\underline{0.00}$	$\underline{8.12}$	$\underline{0.00}$	$\underline{8.42}$	$\underline{0.00}$
Thunderbird	2	0.49	0.92	0.37	$\underline{2.06}$	$\underline{0.05}$	$\underline{5.43}$	$\underline{0.00}$
Gault	2	0.35	0.15	0.88	0.60	0.55	$\underline{2.98}$	$\underline{0.01}$
Gault	3	0.35	0.06	0.95	0.34	0.74	1.52	0.14

Yet pairwise comparison of datasets did not always pattern consistently. Regression slopes for Callahan Stage 3 and Adams Stage 2 were nearly identical, as were slopes for Callahan Stage 4 and Adams Stage 3 (Table 6). That is, Callahan Stage 3 patterned in slope as did Adams Stage 2. Callahan Stage 2 had a higher slope than any other regression, and Callahan and Adams data did not correspond directly by stage, Adams’ values instead being offset by one stage relative to Callahan’s. Generalizing from this pair, Callahan’s Stage-2 slope coefficient was considerably higher than most other datasets’ (Pelegrin and Chauchet’s Stage 2 excepted) and values for its successive stages often approximated those for other datasets’ next-lowest stage. Possibly Callahan data differed in some unrecognized way from other sources, or the large Callahan sample and its wider range of technological and size variation, roughly encompassed by its three stages compared to one or two in other sources, produced the difference. Alternatively, ambiguity in Callahan stage assignments accounts for the difference.

Whatever the cause, this difference suggests unexplained variation between datasets. To interrogate this variation, the Callahan dataset’s wide range of “stage” assignments and variation justifies its examination first. Separate PCA and variable standardization were performed on Callahan Stages 2–3 and Stages 3–4 combined in Callahan data alone. That is, only preforms in Stages 2–3 were submitted for z-score PCA analysis, then separately only preforms from Stages 3–4 were analyzed. This treatment approximates the two-“stage” range of other datasets. If the separate subdivisions of Callahan data pattern and scale with reduction as do other datasets, then differences between datasets owe to Callahan’s wider range of data variation. In both Callahan subsets, z-weight was regressed against z-PC1 by stage. In Callahan’s complete dataset, three slope coefficients ranged from 0.69 to 1.37 (Table 7). In the subsets of combined Stages 2–3 and combined Stages 3–4, four slope coefficients together ranged from 0.79 to 1.23, a modest reduction. In each paired subset, the two successive stages differed significantly in slope (in the Stage 2–3 subset, t = 2.17, p = .04; in the Stage 3–4 subset, t = 2.03, p = .05), as they did in the complete Callahan dataset. However, slope coefficients did not differ significantly by stage between Callahan’s complete dataset and paired-stage subsets (Table 7). Variation in Callahan’s data is reduced only modestly by this treatment.

**Table 7:** Summary of test of similarity in B1 coefficients between original Callahan data and separate Callahan combinations of Stages 2–3 and 3–4.

Stage			Callahan	Stage 2	Callahan	Stage 3	Callahan	Stage 4
Subset	stage	B1	t	p	t	p	t	p
none	2	1.37						
none	3	0.94						
none	4	0.69						
2&3	2	1.23	0.65	0.52				
2&3	3	0.84			0.70	0.49		
3&4	3	1.01			0.43	0.67		
3&4	4	0.79					1.58	0.12

Congruence in slope by stage was inconsistent in separate analysis of other datasets, although Callahan and Pelegrin and Chauchat Stage 2 sets were similar, as was significant difference between stages. For instance, Adams Stage 2 had a significantly higher slope than did Adams Stage 3 (Table 8); although Gault Stage-2 preforms had a higher slope than did its Stage-3 ones, that difference was not significant. Also, although Adams Stage 2 and Stage 3 differed significantly in slope, Adams Stage 2 and Gault Stage 3 did not, despite the greater absolute difference in slope coefficients.

**Table 8:** Summary of test of similarity in B1 coefficients among data sources, excluding Callahan. Italics and underscores indicate t and p values significant @ .05, also shaded for ease of reference.

		z-weight		z-weight		z-weight		z-weight		z-weight		
			Adams 2		Adams 3		Thunderbird		Gault 2		Gault 3	
Dataset	stage	B1	t	p	t	p	t	p	t	p	t	p
Adams	2	1.02	--	--								
Adams	3	0.69	$\underline{2.36}$	$\underline{0.03}$	--	--						
Thunderbird	2	0.97	0.42	0.68	$\underline{2.73}$	$\underline{0.01}$	--	--				
Gault	2	0.89	1.04	0.31	1.59	0.14	0.77	0.45	--	--		
Gault	3	0.54	1.55	0.13	0.75	0.47	1.80	0.09	1.23	0.24	--	--
P&C^1^	2	1.30	1.37	0.18	1.65	0.12	$\underline{2.23}$	$\underline{0.04}$	1.36	0.19	1.10	0.29
P&C	3	0.62	$\underline{3.55}$	$\underline{0.01}$	0.86	0.41	$\underline{2.40}$	$\underline{0.02}$	$\underline{3.57}$	$\underline{0.02}$	0.42	0.68
P&C	4	0.18	$\underline{3.52}$	$\underline{0.01}$	$\underline{12.12}$	$\underline{0.00}$	$\underline{4.29}$	$\underline{0.00}$	$\underline{3.55}$	$\underline{0.00}$	1.99	0.07
		JTI		JTI		JTI		JTI		JTI		
			Adams 2		Adams 3		Thunderbird		Anzick		Gault 2	
Dataset	stage	B1	t	p	t	p	t	p	t	p	t	p
Adams	2	0.39	--	--								
Adams	3	0.18	$\underline{1.78}$	$\underline{0.09}$	--	--						
Thunderbird	2	0.49	1.69	0.10	0.83	0.42	--	--				
Gault	2	0.35	0.36	0.72	0.74	0.48	0.68	0.51	0.87	0.40	--	--
Gault	3	0.35	0.12	0.91	0.57	0.58	0.39	0.71	0.74	0.47	0.03	0.98

^1^Pelegrin & Chauchat

More robust patterning might be found by pooling data by assigned stage from all datasets. This treatment has the added virtue of attenuating the Callahan dataset’s wide range of variation. In all datasets combined, results are clear (Fig 14a):

Combined Stage 2 *z* − *weight* = 0.01 + 1.05 * *z* − *PC*1 Eq. 1

              (.06) (.05)

Combined Stage 3 *z* − *weight* = 0.02 + 0.82 * *z* − *PC*1 Eq. 2

              (.06) (.06)

Combined Stage 4 *z* − *weight* = −0.14 + 0.64 * *z* − *PC*1 Eq. 3

              (.04) (.04)

Results are significant for several reasons. First, Stage 2 preforms patterned in z-weight against z-PC1 essentially at unit rate (i.e., B1≈1), successive “stages” at proportionally-lower rate. That is, early segments of the reduction continuum involve unit reduction in weight and volume, while later segments progressively decline in weight reduction relative to volume reduction. Accordingly, B1 serves as an allometric coefficient that declines steadily as reduction advances. Second, in the combined dataset—though not necessarily in individual ones, as separate analysis above demonstrated—“stages” apportion this allometric variation fairly proportionally, B1 coefficients declining by nearly equal magnitude by stage. Third, all stages’ B0 coefficients →0, although Stage 4’s is slightly but significantly negative. Therefore no constant, either positive or negative, is needed to account for variation between stages. Magnitude of decline of z-weight with z-PC1 was roughly constant in subsequent assigned stages, as successive B1 values were evenly spaced. Finally, all successive stage pairs differed significantly in B1 (between stages 2 and 3, t = 2.42 p = .02; between stages 3 and 4, t = 2.44 p = .02).

Unlike analysis of separate datasets, that is, pattern and scale both were consistent in combined data. B1 consistently declined as reduction advanced, and rate of decline consistently declined by near-constant magnitude across stages. Whatever the differences in pattern and scale by original sources, combined data are consistent in both respects. Differences in stage assignment between datasets effectively cancel out in combined analysis, where more robust results are consistent not only in pattern but also scale.

#### JTI

The JTI index patterned with PC1 somewhat more ambiguously (again, JTI was not computed for Pelegrin and Chauchat replicas). No JTI slope coefficients were statistically similar to their corresponding stages in Callahan’s own data (Table 6). Gault Stages 2 and 3 were identical in slope but different in intercept or location. Excluding Callahan data, the only near-significant differences in JTI slope upon z-PC1 were between Adams Stage 2 and its Stage 3 (Table 8). Otherwise, JTI slope coefficients did not differ significantly although in all cases Stage 2 values always were ≥ Stage 3 values, and Stage 2 values ranged from 0.35 upward, Stage 3 from 0.14 to 0.35.

In all datasets combined, results were as follows (Fig 14b):

Combined Stage 2 *JTI* = 1.81 + 0.38 * *z* − *PC*1

           (.06) (.06)

Combined Stage 3 *JTI* = 1.59 + 0.22 * *z* − *PC*1

           (.05) (.05)

Combined Stage 4 *JTI* = 1.24 + 0.07 * *z* − *PC*1

           (.03) (.04)

Difference in Stage-2 and -3 slopes approached but did not reach significance (t = 1.75, p = .08) but Stage-3 and -4 slopes differed significantly (t = 2.25, p = .03).

## Discussion

Continuous measures z-weight and JTI *patterned* with overall preform size as given by z-PC1. At successive Callahan “stages,” most regression slopes against z-PC1 declined. That much is consistent. However, individual datasets did not *scale* similarly because the same Callahan “stages” sometimes yielded significantly different z-weight slope coefficients. That much is ambiguous. In particular, Callahan’s own dataset differed significantly in z-weight slope coefficient from most others, which in turn scaled nearer to one another.

Why the scale ambiguity between datasets? One possible reason is that the technological criteria by which Callahan stages are defined are independent of size and continuous measures of reduction like weight and JTI. Another is that stage assignments and the criteria on which they are based are not replicable between analysts. As Dickens [18] suggested, one archaeologist’s Stage 2 may be another’s Stage 3. Complex patterns of both discrete and continuous variation are imperfectly captured by “stage” assignment, and when using stages datasets may not scale consistently even if they pattern clearly.

Among other things, the scale differences between individual datasets suggest that none, including Callahan’s, is an ideal master sequence against which future data should be compared. Fortunately, results of combined analysis of all datasets by stage assignment were consistent in both pattern and scale and, because Eq. 1–3 B1 values were fairly evenly spaced, implicate constantly declining rate of decline of z-weight upon z-PC1. Results support a continuous approach to both the reduction process and its analysis. They also suggest that the ambiguity in stage assignment noted above is offset in larger, combined datasets. To this extent, combined datasets like the final one analyzed here are better reference datasets for future studies. In general, JTI results were similar but statistically less robust. Further research is needed to determine how well JTI performs in reduction analysis.

Based on this analysis, the slope coefficient from regression of z-weight upon z-PC1 conveys useful information. Calculated easily in SPSS or other common platforms for any preform assemblage that reports length, width, thickness and weight, it can aid future studies in several ways. For instance, possible stage assignments in other preform assemblages, particularly relative homogeneous ones like caches, can be tested by analyzing them as here; assignments are validated if they approximate the regression results of Eq. 1–3. For combined datasets, that is, results scale the various stage assignments found in the literature to a single reduction continuum. If datasets analyzed here are representative--a plausible tentative hypothesis considering the range of toolstones, contexts, and empirical and experimental sources encompassed--they comprise a reasonable continuum of biface-production variation between original blanks and nearly finished tools, by which ambiguous, ordinal-scale stage assignments can be more precisely calibrated. Even more useful is to abjure qualitative stage designations and instead analyze relatively homogeneous subsets of larger preform assemblages.

Such assemblages (e.g., from caches), can be analyzed as here. Resulting regression coefficients then can be used as continuous measures of degree of reduction. Coefficients would be particularly useful in detailed models of biface reduction across the landscape, including behavioral-ecology ones (e.g. [33–34, 66]), thereby resolving apparently qualitative production stages or steps to the continuous process that underlies them. For instance, instead of assigning, say, two preform assemblages to Callahan Stage 2 and a third to Stage 3, analysis could demonstrate that one lies at the point where z-weight’s B1 coefficient upon z-PC1 is, say, 1.07, a second at 0.94 and a third at 0.61. In this way, all three can be situated at different, relatively precisely calibrated segments of the reduction continuum modeled by Eq. 1–3, and measure biface reduction across landscapes at scales commensurate with continuous behavioral models (e.g. [34, 67]). Similarly, preform assemblages that can be subdivided by context, toolstone or technological criteria can be compared and contrasted for the segments or positions in reduction continua that regression analysis indicates.

The stage concept remains an ambiguous descriptive and perhaps analytical heuristic until a sufficient set of valid, replicable and usually discrete technological criteria can be specified. They must be valid in the sense that the theory of brittle fracture and/or detailed replication experiments demonstrate the criteria’s contribution to the patterning in data by which stages emerge. They must be replicable in the sense that all analysts who apply them to the same experimental or empirical preforms would, as a result, make the same stage assignments.

At the same time, continuous reduction measures like weight, JTI, FI and others (e.g., 47–49) should be recorded on the same replicated specimens. One promising area of research is scanning methods that might make efficient and rigorous the measurement of cross-section variables like area and symmetry and faceting variables like number, pattern and size variation in facets (e.g. [68–71]. If such measures covary strongly with levels or states of qualitative technological criteria and if they contribute to the definition of relatively cohesive groups of specimens distinguished from others by considerable distance in bivariate or multivariate space, then they corroborate the value of the stage approach. Measures may not covary strongly with levels or states of qualitative criteria but instead pattern independently of them, as significant covariation between weight and JTI on the one hand, and size as measured by PC1 on the other. In that case they corroborate the independent value of continuous measures and cast doubt on the validity of stages. If so, we should use controlled replications to define entirely continuous reduction models that efficiently summarize multivariate patterns in dimensions, weight, gestalt size as measured by PC1, JTI and other reduction measures.

Until then, the most conservative approach is dual. For comparison to earlier studies, and for somewhat ambiguous descriptive convenience, the interpretive construct “stage” may be justified. Yet it is essential to report measures like size, weight, and JTI and to analyze continuous variation in them. At least crudely, the two approaches can be compared and possibly cross-validated by compiling the proportion of preforms assigned to Callahan stages and also plotting z-weight or JTI against z-PC1. Within assigned “stages,” regression slope coefficients might approach values found here for the comparable “stages” either in Callahan or other datasets. Across them, pattern in slope coefficients should approximate those found here. As proportions shift progressively toward more advanced reduction, slope of regression of z-weight or JTI upon z-PC1 should decline. This possibility can be tested in the comparison of preform assemblages from quarries to workshops or residences [34], between time periods, or otherwise across space as the reduction continuum can be segmented. As much as possible, such analyses should control for toolstone, time-space, and industrial differences.

Even if stage and continuum approaches are complementary, the latter possesses some unique advantages. By definition, continuum models document fine-scale variation in and between assemblages. Such variation can owe to comparable adaptive or technological variation, or chronological or cultural change. Continuous variation also can be interrogated for sometimes fine-grained conformity to continuous-scale behavioral models [34, 67, 72] in ways that coarser stage models cannot.

Although preforms in the reduction process were the focus of this study, of course finished bifaces continued to be reduced in use for resharpening and repair. Archaeologists have devised a range of measures at the level of individual specimens to track the allometric variation produced by these practices [47, 54, 58, 73–76], as well as variation that is not allometric [77]. It is worth examining assemblage-level variation in finished points by such measures to complement the assemblage-level variation in reduction preforms documented here, mindful that different measures can be suitable for preforms and finished points.

## Conclusion

None of this is to criticize biface-production stage approaches generally, nor to suggest that reduction is continuous in all salient respects. Callahan’s ([1], Table 10) model includes categorical variables that may pattern with assigned stage. Unfortunately, some are difficult to replicate or of unknown relevance (e.g., regularity of outline, “degree of concentration during fabrication,” “degree of trim,” “nature of reduction emphasis” which in Hill’s [[11], Table 1; see also [19], Table 5–23] approach signify relative emphasis upon edge, thinning, and outline, however determined).

Callahan’s and other traditional approaches to the reconstruction of reduction sequences are highly detailed. Yet stage models are not always valid and replicable, nor do they control a considerable dimension of continuous variation. There is robust patterning in the relationship between continuous preform variables of weight, JTI and linear dimensions that would not emerge from a stage approach [16]. Complete analyses of any reduction sequence may be qualitative to some extent but also must be quantitative, for both individual specimens and entire assemblages. This paper suggests some statistical methods for continuous data, possibly useful in reduction-sequence analysis, that complement detailed technological description.

## Supporting information

S1 Table Stage assignment, dimensions and weight for study preforms. (XLSX)
